# Fifty-Years’ Experience in Obstetrics

**Published:** 1891-08

**Authors:** John S. Clark

**Affiliations:** Chicago, Ill.


### FIFTY YEARS' EXPERIENCE IN OBSTETRICS.*

By JOHN S. CLARK, M. D., CHICAGO, ILL.

MR. PRESIDENT AND GENTLEMEN—When I set out to write these my obstetrical reminiscences and conclusions, in a rather disjointed manner, as I could catch time in the intervals of pro-

*Paper read before the Chicago Gynecological Society, February 20th, 1891.

fessional business, it seemed quite a long and, as I thought, rather a disagreeable article, going much into detail; and it was long and dry and tedious. I had not gotten half through with it when I remembered how much I had suffered in acquiring the knowledge and facts set forth, and I felt I had no right to inflict upon you gratuitously what I had endured and been paid for; so I set about, rather late in the day, to summarize and shorten my article, and found the last day, and in the afternoon, before it was two-thirds finished.

This is the Golden Age of the obstetric art. When Zeuxis and Apelles were painting those wonderful pictures which deceived birds and animals, and Phidias and Lysippus carving those colossal statues of gold and ivory, many of which were sold for their weight in gold—at that time, which has been by many considered the Golden Age of the fine arts, one art was far behind.

Hippocrates, who was a contemporary of these artists, begins his celebrated aphorisms with: "Life is short and the art long; the occasion fleeting, and experience fallacious, and judgment difficult. The physician must not only be prepared to do what is right himself, but also to make the patient, the attendants, and externals co-operate."

I quote the whole of the first aphorism, so wisely, so nobly expressed. But our art was then in its infancy, comparatively, as I will illustrate by another quotation from the same great man: "When the child presents double at the mouth of the womb, it should be pushed upward so that the head may come down.

"When a hand or foot protrudes it is to be pushed up in like manner, and the head made to present."

When, in feet presentations, the head is retained after the body is delivered, he advises us "to introduce a hand between the *os uteri* and the head, and deliver it."

When the secundines are retained, he orders us "to extract them slowly," and for this purpose directs that, the woman being placed on a stool, the child not having been separated, it is to be allowed to hang down, so that by its weight it may produce separation; and, "lest its weight should occasion too strong pulling," he advises "it be laid on wool, or bladders filled with water, so that when perforated the child shall sink down gradually and draw away the placenta."

Celsus directs us, "in arm presentation, to pull down the head with a hook in the eye, the ear, the mouth, or forehead."

Aetius gives as a cause of difficult labor, "A too compact union of the *ossa pubis.*"

According to Eros, difficult labor is due to "tumefaction of the external parts," for which he advises "a sitz bath prepared with emollient herbs."

Avicenna states that the expulsion of the child is performed by the abdominal muscles, and this was the opinion of Galen. He directs baths before and during labor, and advises the "use of forceps in difficult labor, the child to be extracted by them." "This," says Francis Adams, the translator of the works of Hippocrates, "proves that the Arabians were acquainted with the use of the forceps."

Haly Abbas mentions imperforate hymen among the causes of difficult labor. Baudelocque reports such a case, so does Burns, and I too have had a case; of course the hymen was not imperforate, but was unruptured.

Haly Abbas directs, in rigidity, to "make the woman sit in a warm bath prepared with chamomile, etc., and to take internally an infusion of swallows' nests"—probably the edible birds' nests so valued by the Chinese.

So much for ancient midwifery. Nor did it improve much through nearly two thousand years. With the discovery of the art of printing began a new era in the arts and sciences. The ease with which one able man and close observer transmitted his knowledge to his fellow-men, and he in turn to his followers, thus exciting a noble emulation, soon brought our art rapidly forward, and we have works, written a hundred years ago, varying but little, and in minor details only, from our present and almost perfect state.

To this great teacher and best illuminator, the art of printing, discovered about 1450, almost coeval with the discovery of our country, and which has done for the minds of men what our land has done for their bodies, do we undoubtedly owe our rapid and brilliant advancement in the arts and sciences, more especially our own art, which has kept so well up in the race that it is now considered well-nigh perfect. It makes me proud to read such a book as that, say, of honest James Blundell, with his repeated warning against "meddlesome midwifery"; to read his direction for the management of everything that may happen to you as an obstetrician, and to know by your own experience that everything he says is true, his advice pure gold. He lectured eighty and more years ago.

Denham, too, so reliable and satisfactory; Cazeaux, one of the most complete manuals ever printed—you never look in him in vain; Velpeau, valuable for the neat manner with which he gives us the benefit of the enormous experience of those wonderful women, Mesdames Boivin and La Chapelle. The lively Gorch, and the sound, reliable, painstaking Ramsbotham, and in this country the patient, indefatigable Dewees, the brilliant Meigs, and last, but not least, the invaluable Lusk—these books, glorious monuments to their authors, better than "storied urn," stand on the shelves of our libraries or lie conveniently at hand on our office tables, generally well thumbed; and we all know that they made us, and we owe them to the press—the printing press.

During the early years of my professional life I went to every case of childbirth with dread and fear of an impending calamity. I constantly read the dear good writers upon the subject, and faithfully followed their teachings. I was watchful, patient, and tried not to be meddlesome. Years of success gave me confidence, and I have come to be, perhaps, too far the other way—too easy and sanguine; but I never go to a case, even now, without something of the feeling of a man going to jail—a man going to be "confined" himself. The leaving of a pleasant home for an uncertain time, the dropping of every other pursuit, the going to reside in the abode of anxiety, uncertainty, and misery make the life of an accoucheur one of great self-denial and often of downright physical and mental endurance; and yet the happy ending of a bad case, "mother and child as well as could be expected," is a most delightful experience and pays for all.

I graduated in January, 1843, at Geneva, N. Y., and, with the exception of a three months' trip to Europe in 1855, have been constantly busy in the practice of my profession ever since. During that time I have attended, in round numbers, three thousand five hundred cases of childbirth. I never saw a woman die in actual labor, and was never called to a case that I left undelivered.

I divide my time into three periods—the first of thirteen years, while a resident of a thriving and beautiful town of some five thousand inhabitants in Central New York, where I attended seven hundred cases, none of which were of sufficient interest to be worthy of especial mention. No placenta previa, no arm presentation, no eclampsia—two face presentations being the most troublesome I met with in those days. I was shy in using forceps, having too much trouble in making them lock, not having then learned the trick of depressing the handles. I only recall three cases in which I used them. It was there I learned how nature, if given time, would overcome what seemed insurmountable obstacles, moulding and shaping the soft, yielding head till it would travel through a strait at first deemed impassable. I had but one death there, from what I now know was uremic poisoning. It occurred thirty hours after labor.

In this city, from 1856 to 1871, I had quite a large obstetrical practice. All records were burned in our great fire, but I am sure I place the number low enough at fifteen hundred cases. In 1857 I had my first arm presentation—a midwife's case, who had dallied with it all day—but kind nature, as she most always does in preternatural presentations, had withheld hard pains, and the turning was easy and quite successful.

The bugbear of my existence had been for years placenta previa, and one stormy, dismal night in March, 1859, I found myself confronted with such a case in a remote place in the Rolling Mill district. There was much flow and a small, rigid os. I tamponed at once with extra care, and sent a messenger for

Dr. Clark, of South Halsted street, a capable, reliable man of the old régime, lately deceased. Dr. Clark had attended the mother with her previous children, and when I explained that the present was one of the most dangerous incidents that could befall a poor woman during child-bearing, the family wanted him sent for, and so did I. After a few hours the tampon began to leak badly and the pains were severe. The doctor had not yet arrived, but so much blood had been lost I dared not to wait longer, so removed the tampon and found an easily dilated os which readily admitted my hand, the placenta barely covering it. The turning was easy, my arm preventing the escape of the waters. The child was dead ; and I will say now that, out of eight cases of placenta previa which I have attended, I have delivered but two living children. The next case of the kind followed this one in a few months, and is only interesting from the fact that, being a midwife's case, she had risked it until the head, pressing pass the placenta, had checked the flow and the labor proceeded naturally. I have attended a lady twice with placenta previa ; in both cases turning was easy, but in the last the patient lingered three weeks and died. I was not able to define satisfactorily the cause of her death, nor could the eminent counsel who saw her with me.

Of arm presentations I recall seven cases ; all turned easily, but the death rate of the children was high ; either three or four died.

For the next period, from 1871 to 1891—twenty years—I have my visiting lists, and from them I gather that during that time I attended thirteen hundred and odd cases, of which a disproportionate number were instrumental. I have for many years been called upon by German midwifes in my neighborhood to deliver their bad cases, or extract adherent placentas, or turn out clots in internal hemorrhages, and this should give me a broader margin of percentage for losses ; and yet I shall not claim it, for there has actually been no loss. As I said before, I never saw a woman die in childbirth, and I have often asked my professional brethren if they had, and almost always the reply is in the negative. But I have had three deaths within twenty-four hours after labor—one at six hours, from exhaustion following a breech presentation. The patient, a very unhappy young widow, pretty and fat, tired of life, would not make an effort, and the labor, a dismal failure all the way through, was finished at last by a blunt hook, leaving her completely exhausted. She could not rally, and died at the end of six hours. The next fatal case was an arm presentation; the woman a poor, dissipated, broken-down creature, who, with a midwife and attendants much like herself, had been in labor all night. I saw her at noon, and had no trouble in turning and delivering, and left her quite happy at being out of her misery. I found, in the morning, that she had died at daylight, dropping off so easily that it was believed she had fallen asleep. The third case I do not remember so well, but think it was simply a tedious labor. The patient died twenty-four hours after delivery. They were all three simply cases of exhaustion. These, and the uremic-poisoning case in New York and four other cases—one of which was a most interesting case of pyemia, in which death occurred thirty-five days after delivery, and which was worthy of a long and full report—are the only ones of death from the dangers of gestation or delivery that I can at this time recall in my whole experience.

Twice I have felt compelled to use perforation. The first time was in a frontal presentation, when, for some cause, we could not make the forceps hold. The next was in a vertex presentation, with face in the hollow of the sacrum. It was a curious case—a heavy, stolid German woman of forty. She had been delivered of three stillborn children, and I had had the good luck to deliver her of a large, fine living child—forceps delivery—two years before. In her fifth confinement the presentation was good, and, after waiting long and giving her a fair chance, I applied the forceps. If I had tackled the Rock of Gibraltar I could not more signally have failed. Then I thought of the best doctor for physical strength within reach, and he was sent for. He tugged away till he was tired out, when the family remembered a remarkably skilful "little" German doctor, who, after a short trial, concluded he could not do it, and we put our heads together and settled on craniotomy. The family would not consent, and another eminent and stouter German doctor was sent for, and he bothered us for hours, trying and resting, and trying again. He finally gave in, and we settled down to the perforator. The baby had been dead for hours. After reducing the head to the smallest dimensions possible with the cranioclast, we could not move it. We removed the frontal and parietal bones, and by that time there was a slight gain, and after mutual efforts, that lasted altogether three hours from the time I sat down with the perforator, the brave patient was delivered, nor was there any very serious trouble afterwards. She was in bed three weeks, but has never conceived again.

I cannot recall a single death or serious harm following a forceps delivery. Since learning to use them neatly, I apply them earlier than I used, but always give Nature a good chance. In my midwife cases I satisfy myself that they are necessary before using them. I often have patients who cry out for them when I enter the house. I use them slowly, imitating nature. I do not consider an hour or two out of the way at all, and I have had them on and off for a day or two, once or twice, with a perfect recovery of the mother.

I have been peculiarly fortunate in regard to hemorrhages after delivery, never but once knowing that my patient's life was in great danger. It was a most interesting and instructive case, but too long for this article. Only once in my entire experience have convulsions seized my patient during labor, or before or after. She recovered, the baby stillborn during coma. I have seen a dozen or more cases in consultation ; at one time I had seen seven consecutive cases that had recovered. I have much faith in venesection, but there are cases I would not bleed. I have not been a strenuous advocate for hurrying delivery ; Nature almost always delivers. That it is a "*sine qua non*" is absurd.

I have had two cases of encephalic monsters ; they did not disturb the course of events.

Spina bifida has been a rare event to me, two cases only that I recall.

I used anæsthetics in labor much more frequently formerly than now. I fail to see their value in most cases, and only use them when the os is, from any cause, very tender and sensitive, or when I am about to undertake a painful obstetrical operation. I have patients who insist upon their use, and in such cases yield gracefully and do the best I can. If not carried to complete anæsthesia, the use of them does not often interfere with the progress of a case, and serves to divert the patient from a too complete consciousness of her pain. But, owing to the varying susceptibility of women, it is not always possible to stop at exactly the right point, and quite lately I have lost a child, a fine, stout boy, from my patient passing to complete anæsthesia (in an instrumental case) the moment the baby's head passed the external soft parts. Now, while it is allowable to make tremendous traction with the forceps upon an undelivered head—the shoulders easily following the head—after the head passes the vulva the situation is completely changed. The shoulders now have to overcome the resistance of the soft parts, and, unless our efforts at extraction are backed up by good, strong, expulsion pains, it cannot be done by any amount of tension we may safely apply to the head, and the blunt hook is the instrument we have to depend upon. In this case my patient was noisy and troublesome till the head was delivered—having had but a few drops of chloroform—but the moment the head was delivered she became completely insensible. The child, a very large one, lay face downward, and the sphincter caught him as neatly as any garroter could have done ; he gasped for breath two or three times, and though I had a good blunt hook at hand and quickly applied it, at the same time trying to take off the compression of the soft parts, did not succeed in delivering until the child was hopelessly gone. If I had had another blunt hook, with the two I could have delivered sooner. I had delivered this woman three times previously, with forceps, of living children, but without chloroform.

I have used ergot quite frequently ever since I began to practice midwifery; at first in inertia only, viz., when pains were feeble; of late years for other purposes, principally for hemorrhages. I have never seen harm result from its use, save in one case over forty years ago. In a tedious case, with the os well dilated, the ergot acted most violently. I have never seen anything like it since. The child was stillborn, and no doubt the use of the forceps would have been much better. Midwives having too much to do use ergot constantly to hurry their cases; and if you happen to get a patient formerly attended by a midwife, the chances are that your case will make but little headway until you use it, the patient having acquired what may be called the ergot habit. I have seen dozens of such cases, using ergot for insufficient pains; one should have the forceps at hand. But why use ergot in such a case at all ? The forceps skillfully applied is safer for mother and child. I never knew the mother to die after a forceps delivery, and very few children. I am sorry I am not able to say exactly how many, but I am sure I have not lost a baby that way for years, except the one just mentioned as lost through the use of chloroform during the instrumental delivery.

I have attended a lady with all her children, ten in number. The first seven were under ten pounds weight, easy, natural labors. Then the mother grew fatter, and the last three children weighed fourteen or fifteen each, with long, tedious instrumental labors. In the last one, the largest child and the most tedious labor, the child stillborn, I was over an hour in delivering the shoulders.

I have delivered a few times from above the superior strait; cannot say how many times; can recall two instances and know there were more.

It would not harmonize with Nature's perfect work that a woman, in carrying out the principal object of her existence, the continuance of her species, should lose her life in giving birth to her child. Our artificial, luxurious mode of living, our refinement and cultivation, and development of the sentiments and emotions, and fineness of figure and fibre, render our women more liable to danger and disaster in child-bearing than when living plainer. But, fortunately, modern science has given the physician almost perfect methods of relieving her from nearly all the ills that female flesh is heir to, and I have a conviction that there is nothing that can happen to women in which there is so little danger as child-bearing. And if women were taught this wholesome truth it would entirely change the color of their lives. Another conviction is that the pains of childbirth are enormously exaggerated in the vast proportion of cases. I have noted this fact in a great number of cases, and nearly all the ladies, when asked about the amount of pain, at the time have said it was much less than they had expected, and a few, a very few, have said "it is nothing at all." But there is this curious contradiction, that, while willing to speak lightly of the pain at the time of labor, when asked about it afterward all have said, without exception, that it was simply awful. So that it seems their testimony cannot be relied upon, and we have to draw our own conclusions; and, as I said before, from what I have seen I do not believe that in the majority of cases there is such severe pain as is usually supposed; and if we can make young women believe this too, we shall brighten the complexion of ther lives and lessen the number of cases of abortion, of which I am about to speak. But before doing so I wish to bear testimony again to the value of the early and prompt application of the forceps in cases of threatened, tedious, wearying labor. They are but a pair of thin, elegantly made steel hands, which, backed by strong arms and skillfully applied, do better service in the cause of women than ever did those bright Toledo blades that cut such a figure in the annals of chivalry. For both mother and child we may say, as Sir Walter Raleigh said of the axe used to behead him, "It is a sharp medicine, but a quick cure for earthly ills."

### ABORTION.

As nations grow more powerful and prosperous, and individuals devote themselves to the getting of wealth that they may lead lives of luxury and pleasure, a disposition to regulate the size of the family prevails, and the slaughter of the innocents begins; so we may say that abortion, like the free use of salt, marks a high degree of culture and civilization. It is not necessary to say anything of the causes of abortion, which are as various almost as the cases. But I have had opportunities of seeing some curious and interesting results following "criminal abortion" which will interest you, and I will mention them.

In several instances fine, healthy, handsome young women, quite recently married, found themselves in the family way earlier than suited their views, and caused criminal abortions to be procured. It was interesting, years afterwards, when they desired to be treated for sterility, to learn from them the story of their folly. They had lost the ability to conceive. It was as if violated Nature required years to recover her propriety.

In two of the cases the mothers of the young women had not only sanctioned the crime, but had gone with them to the abortionist. Many years ago I was the physician to a lady, mother of three healthy children, who became one of what I may call a colony of abortionists. A cultivated and accomplished young married lady with one child had moved into the neighborhood, and soon taught a number of the ladies, her more intimate friends, the art or trick of rupturing the membranes with a goose quill. My patient was one of the initiated. I will not attempt to say now how often I attended this lady with her abortions in the few following years; she had acquired the habit, and abortions would recur in spite of her. As soon as she discovered this it became the end and aim of her existence to have another living baby; it was years before she succeeded, then in less than a month the baby died with convulsions, and another and another succeeded, each succeeding one attaining greater age and the mother proportionately more fond. It was the most pitiful sight I ever saw, this anxious, pale, sad-faced mother watching those delicate children as they drooped and died. Finally one lived, a sturdy fellow, who as a child was the terror of the neighborhood, as a young man was a thief and could not be trusted in any way. In such manner may geniuses for good or evil be made. The lady who so thoughtlessly corrupted her friends is long since dead, though descended from a long-lived race. She was ambitious and desirous of a social position; they had been poor, but worldly matters went well with them, and she might have been living now, a happy grandmother, but for this most unhappy turn in her affairs.

I am not able now to say how many cases of abortion I have attended. If I put them at twenty a year it would make the number a thousand; but when I say I have had four cases in the past month, and have always had a good number, it would not, at all events, be out of the way to say six hundred cases. I believe there were more—well, say six hundred; and when I add that I never saw a woman die from hemorrhage in abortion, a valuable and interesting fact is stated, and one which should bring a goodly degree of comfort to the unfortunate doctor who is compelled to attend these doubtful, confusing, tormenting cases. And speaking of hemorrhage reminds me that a former patient, mother of one child, much to her annoyance failed to menstruate at the proper time, and a month or two later, to her great delight, found herself flooding so violently that I was sent for. I put her to bed, enjoined rest and perfect quiet, and gave her an opiate. She lost an immense quantity of blood, large clots coming away; she hoped everything had passed. The same thing happened a month later. I did not see her, but at the proper time, six months afterward, delivered her of a fine healthy child.

It is astonishing how long a time, occasionally, will elapse after the death of the fetus, before it is cast off. I have had a case where three months passed after its death before it was expelled, the patient troubled all the time with a pink show. It is not necessary or desirable that I should say much about the treatment of cases of abortion; we all treat them similarly. I have found the hemorrhage easily controlled by the well-fitting tampon, and have never seen harm from its use. If the ovum is long retained and hemorrhages occur, we should try to get it; for, once the ovum is turned out, the bleeding ceases. I have known the ovum retained twenty-eight days, and finally discharged with very little show, perfectly inodorous and unchanged; and Dr. Hasbrouck, of Nyack-on-the-Hudson, reports a case retained sixty-five days. If decomposition takes place, and there are very offensive discharges, and the os open, and the cavity of the womb easily reached, I should say by all means turn it out; but if the external os is tightly closed, I should hesitate to invade the sanctuary on which Nature has written, "No admittance." From a great number of cases that I have had of retained decomposing ova which came away or were absorbed without harm to the patient, I have come to have little fear as to the result.

In a little book entitled "The Physician Himself," written by Dr. Cathell, of Baltimore, and dedicated to Professor Austin Flint, Sr., I find these golden words :

"When you are importuned to produce abortion, on the plea of saving the poor girl's character, or to prevent her sister's heart from being broken, or her father from discovering her misfortune and committing murder, or to prevent the child's father from being disgraced, or to avert the shame that would fall on the family, or the church scandal, etc., etc., or to limit the number of children for married people who already have as many as they want, or for ladies who assert that they are too sickly to have children, or that their suckling child is too young to be weaned, etc.—you should meet them with a refusal as cold as ice, and never even seem to entertain the proposition. If they are too importunate, express your sentiments strongly." "How could any one but a fool be induced to take the burden from another's shoulders to his own by doing a crimson crime ; to violate both his conscience and the law; to risk exposure, social and professional ruin, and the penitentiary, by putting himself into any one's guilty power, whether as a favor or for a paltry fee ?"

